# Rigid locked antegrade versus retrograde intramedullary nailing in treating acute humeral shaft fractures: a systematic review with meta-analysis

**DOI:** 10.1530/EOR-2024-0136

**Published:** 2026-02-04

**Authors:** Lucía Lanuza Lagunilla, Alfonso Muriel García, Jorge Díaz Heredia, Raquel Ruiz Díaz, Vanesa González Sastre, Miguel Ángel Ruiz Ibán

**Affiliations:** ^1^ Hospital Universitario de Cabueñes, Gijón, Asturias, Spain; ^2^ Hospital Universitario Ramon y Cajal, Madrid, Spain; ^3^ Departamento de Cirugía, Ciencias Sanitarias Y Medicosociales, Universidad de Alcalá de Henares, Alcalá de Henares, Madrid, Spain

**Keywords:** humeral shaft fractures, intramedullary nail, antegrade, retrograde, humerus nailing, meta-analysis, systematic review

## Abstract

**Purpose:**

- There is a lack of consensus regarding the optimal intramedullary nailing (IMN) strategy for humeral shaft fractures (HSFs): both antegrade IMN (aIMN) and retrograde IMN (rIMN) are used. We aim to compare both strategies in terms of outcomes and complications.

**Methods:**

MEDLINE, Embase and Cochrane Central Register of Controlled Trials databases were searched for articles in English or Spanish from inception to Nov 23, 2023. All studies reporting on primary treatment of acute HSFs (OTA/AO 12A, 12B, and 12C) with rigid locked IMN, in patients aged 16 years or older, were analyzed. Two independent reviewers screened studies for eligibility, performed data extraction, and used RoB 2, ROBINS-I and JBI’s critical appraisal tools to assess bias of the included studies. Stata 18.0 software for data analysis was used. Subgroup analysis was performed to explore heterogeneity. We applied GRADE to appraise our evidence.

**Results:**

Nine studies involving 565 fractures were included. aIMN showed significantly faster operative time than rIMN (MD = −14.40 min (95% CI: −17.75 to −11.04); *I*^2^ = 0%; *P* < 0.001), and rIMN showed significantly higher odds of intraoperative fractures than aIMN (RD = 0.05% (95% CI: −0.08% to −0.02%); *I*^2^ = 0%; *P* < 0.005). In contrast, aIMN showed significantly higher odds of worse shoulder function and pain complaints and worse Neer scores than rIMN (*P* = 0.02, *P* = 0.03 and *P* = 0.02, respectively).

**Conclusions:**

Compared with rIMN, aIMN demonstrated a significantly faster operative time and lower rates of intraoperative fractures. Conversely, rIMN demonstrated lower rates of shoulder pain complaints and better shoulder function and Neer score outcomes.

## Introduction

Nonoperative management with functional bracing has traditionally been the preferred method of treatment of humeral shaft fractures (HSFs) (1). However, some recent meta-analyses (MAs) have shown considerable rates of treatment failure of this technique (1, 2). Lately, there has been a shift in the management of these fractures, with an increasing proportion of operative treatment of HSFs (3). Despite the never-ending discussion on plating vs intramedullary nailing (IMN), nailing is widely used for the management of HSFs with satisfactory results (4).

There is a lack of consensus regarding the optimal IMN strategy for HSFs: both antegrade IMN (aIMN) and retrograde IMN (rIMN) are used (5, 6, 7). aIMN seems to be technically easier but is associated with shoulder dysfunction and potential neurovascular injury during distal locking (8); rIMN, developed to avoid these complications, is more technically demanding and has pitfalls such as posterior cortex comminution and worse elbow function (8).

In 2022, Kumar *et al.* (9) performed a MA on this topic, including both rigid and elastic humeral nails, but the latter are now rarely used in adults as they do not provide adequate rotational stability (10). The purpose of the present systematic review and MA was to compare the outcomes and complications during rigid locked aIMN and rIMN, for the treatment of acute HSFs in non-pediatric patients. We hypothesized that there are no clinically relevant differences between both approaches.

## Materials and methods

This systematic review was conducted according to the Methodological Expectations of Cochrane Intervention Reviews (MECIR) (11) and reported following the Preferred Reporting Items for Systematic Reviews and Meta-analyses (PRISMA) guidelines (12) (Supplementary materials – Table 1 – PRISMA 2020 checklist - Table 2 - PRISMA 2020 for Abstract Checklist (see section on Supplementary materials given at the end of the article)). The protocol for this study was registered prospectively with the International Prospective Register of Systematic Reviews (PROSPERO registration number: CRD42022336234), on June 10, 2022. Institutional Review Board approval was not necessary.

### Eligibility criteria

The inclusion criteria were original studies reporting on outcomes of primary treatment of acute HSFs (OTA/AO 12A, 12B, and 12C) with rigid locked IMN, in non-pediatric patients (≥16 years). The exclusion criteria were as follows: i) studies published in a language different from English or Spanish, ii) studies that included one or more of the following: pathological, recurrent, periosteosynthetic or periprosthetic HSFs, HSFs with metaphyseal extension or non-acute HSFs (surgical delay >40 days), iii) less than 10 cases reported, iv) less than six months of follow-up, v) treatment with methods different from rigid and locked IMN, vi) reports on specific subpopulations of patients (e.g., HIV and polytrauma patients), vii) publication types different than randomized controlled trials (RCTs) and non-randomized studies of the effects of interventions (NRSIs), including observational studies (cohort studies, case–control studies, and case series (CSs)), viii) studies with critical data missing, ix) studies with no availability of full text and x) studies with duplicate data.

### Search strategy

Embase and MEDLINE general databases and Cochrane Central Register of Controlled Trials (CENTRAL) were searched, ranging from inception of each database to November 23, 2023. Search strings were made by two medical librarians by peer review (see Supplementary materials – Table 3 – Literature search strategy).

### Study selection

All references were exported to Rayyan (13), and two independent reviewers (MARI and LLL) screened the titles and abstracts. Any discrepancies were resolved by automatic inclusion in the next stage of screening. A thorough full-text review was then independently conducted by the same two reviewers for consideration on inclusion (the corresponding authors of studies with no available full-text version were contacted once by email to retrieve the full-text). Any disagreements in selecting a final set of articles were resolved through discussion, and with the help of a third reviewer (JDH) when necessary. Articles that had cited the studies included for full-text review were tracked using Web of Science citation mapping, looking for additional eligible studies. All references of the included studies were reviewed for potentially useful studies. Experts in the field were also contacted to identify additional unpublished studies.

### Data extraction and risk of bias assessment

The same two independent reviewers performed data extraction and risk of bias assessment. All discrepancies reached consensus by discussion.

Data were extracted using a self-designed Microsoft Excel (Microsoft Corporation, USA) sheet. We collected the following data: author, publication year, study period, country, study design, sample size, patient demographics (gender and age), open fractures, HSF OTA/AO classification, primary nerve palsies, nail implant, nail insertion point, follow-up rate, mean follow-up, operative times (authors considered in the meta-analysis of this outcome did not define if operative time was measured from skin incision to skin closure or if it includes patient positioning), intra-operative blood loss, fluoroscopy time, intraoperative fractures, time to union, delayed and nonunion (we categorized as delayed union those fractures that healed within four and six months of intramedullary nailing, and as nonunion fractures that healed between the sixth month and upward after surgery, or which had to be revised later for lack of consolidation), malunion (authors did not define the term), iatrogenic nerve palsies, infection (superficial and deep), the presence of heterotopic ossifications, nail complications and reoperations (related to infection, nonunion, nail-related complications and other causes) and functional outcomes, including pain complaints, shoulder and elbow range of motion (ROM) and patient-reported outcome measures (PROMs): Constant–Murley score (CMS), University of California, Los Angeles (UCLA) shoulder score, American Shoulder and Elbow Surgeons (ASES) scores, Neer score, Mayo Elbow Performance Score (MEPS), Broberg and Morrey scoring system and disabilities of arm, shoulder and hand (DASH) score. Raw data were not requested from the study authors.

We assessed RCTs using the Cochrane risk of bias (RoB) 2 tool (14); NRSIs, including cohort studies and case–control studies, were assessed using the Risk Of Bias In Non-randomized Studies – of Interventions (ROBINS-I) tool (15); and CSs were assessed using the Joanna Briggs Institute (JBI) critical appraisal tool (16). Funnel plots and Egger’s tests were planned for exploration of the potentially publication bias when a minimum of ten studies were included (17).

### Statistical analysis

Stata 18.0 (StataCorp, USA) was used for data analysis. A random-effects model was used to pool effects of treatment options and presented as risk difference (RD) on dichotomous variables and mean difference (MD) on continuous variables, each with a 95% confidence interval (95% CI). Outcomes with zero events in both the antegrade group (AG) and retrograde group (RG) in all the studies were excluded from the MA itself. Heterogeneity between studies was judged by visual inspection of the forest plots and quantified by the *I*^2^ index (18). For this meta-analysis, a value >50% indicated high heterogeneity. In addition, a subgroup analysis based on study designs (RCTs and NRSIs) was planned when we encountered heterogeneity. A standard *P*-value of <0.05 was used to determine statistical significance.

### Evidence quality assessment

Following recommendations of the Cochrane methods, evidence quality was evaluated using the Grading of Recommendations, Assessment, Development and Evaluations (GRADE) approach (19, 20, 21).

## Results

### Search results

**Figure 1:**
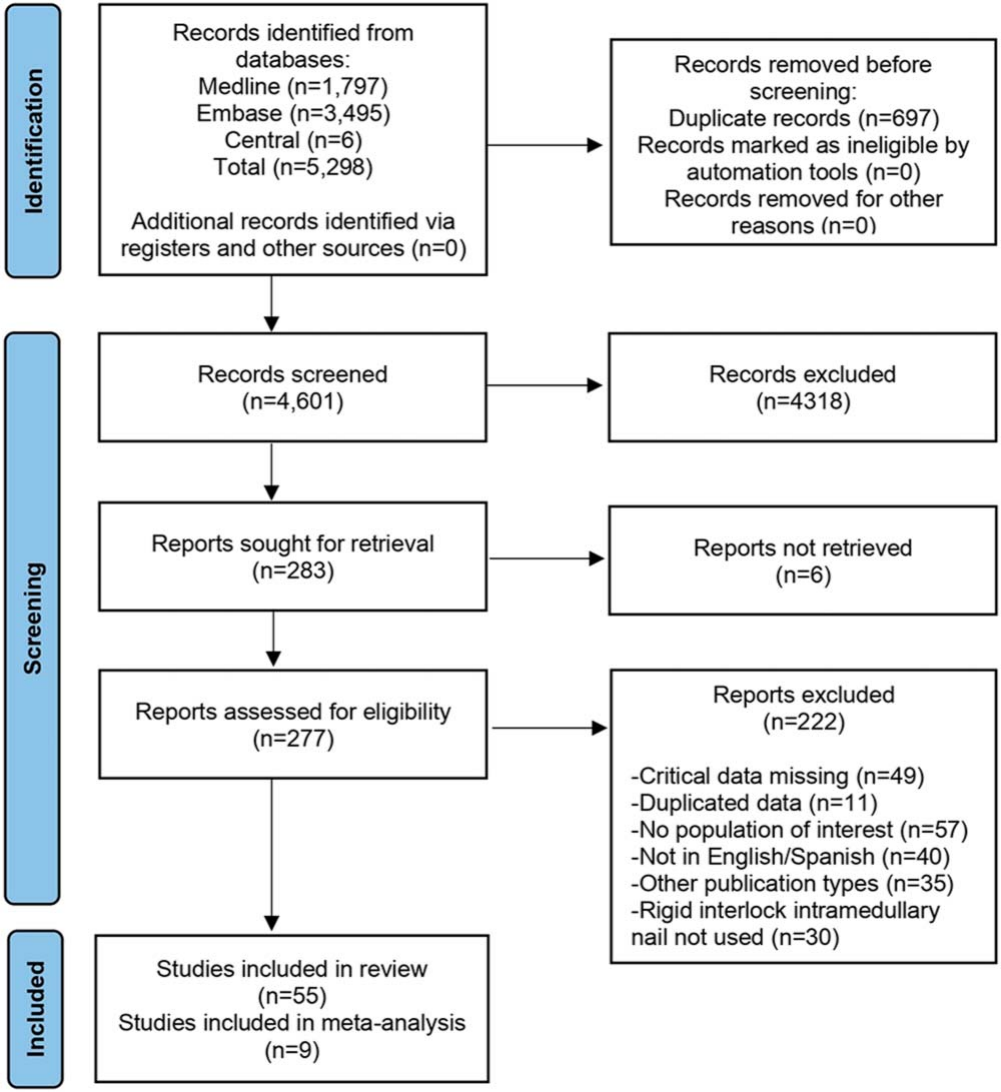
PRISMA flowchart summarizing the search strategy.

The initial literature search identified a total of 5,298 reports, with 283 undergoing full-text article reviews. Fifty-five studies met the inclusion criteria and were included in this systematic review (5, 6, 8, 22, 23, 24, 25, 26, 27, 28, 29, 30, 31, 32, 33, 34, 35, 36, 37, 38, 39, 40, 41, 42, 43, 44, 45, 46, 47, 48, 49, 50, 51, 52, 53, 54, 55, 56, 57, 58, 59, 60, 61, 62, 63, 64, 65, 66, 67, 68, 69, 70, 71, 72, 73) (2,388 patients; 2,394 fractures). Of these, nine studies (5, 6, 8, 22, 25, 32, 33, 34, 46) contained data from both interventions and were included for analysis. Figure 1 outlines the workflow according to PRISMA 2020 (74).

### Study characteristics

A total of nine studies were selected: two RCTs (5, 8), three prospective NRSIs (6, 25, 33) and four retrospective case series (22, 32, 34, 46). In all the studies analyzed in this review, 565 fractures were treated, 562 patients aged 49.35 ± 17.93 years, ranging from 16 to 86 years, although only two studies provided age ranges (22, 32). From all included fractures, 310 fractures were treated with aIMN and 255 fractures were treated with rIMN. The study characteristics and patient demographic data of the studies included in the MA are reported in Table 1.

**Table 1:** Study characteristics and patient demographic data.

Study	Study design	PTS, *n*	Fractures, *n*		Age*, years		Open fracture, *n*		AO type A|B|C, *n*		Primary NP, *n*		Implant		FUR, %		MFU, months	
			aIMN	rIMN	aIMN	rIMN	aIMN	rIMN	aIMN	rIMN	aIMN	rIMN	aIMN	rIMN	aIMN	rIMN	aIMN	rIMN
Blum *et al.* (6)	P/NRSI	84	27	57	NR	NR	NR	NR	NR	NR	NR	NR	UHN	UHN	100	100	Until union	Until union
Cheng & Lin (5)	P/RCT	89	44	45	43.2 ± 19.3	48.3 ± 21.4	8	8	28|13|3	36|7|2	5 + 1^†^	4 + 1^†^	HLN UOC	HLN UOC	100	100	18.6 ± 3.1	19.8 ± 3.7
Ingman & Waters (22)	RCS	20	5	15	36 ± 19.7 (20–63)	36.7 ± 21 (16–80)	NR	NR	3|2|0	13|4|0	1	4	modGK	modGK	100	100	14.6	7.8
Lin (25)	P/NRSI	48	15	33	NR	NR	NR	NR	NR	NR	1^†^	NR	NR	NR	100	100	NR	NR
Metsemakers *et al.* (46)	RCS	125	107	18	NR	NR	NR	NR	NR	NR	NR	NR	UHN, EHN, MHN	UHN, EHN	100	100	Min 12 or until union	Min 12 or until union
Mückley *et al.* (33)	P/NRSI	36	22	14	NR	NR	NR	NR	7|15|0	6|6|2	0	0	T2	T2	100	100	NR	NR
Rommens *et al.* (34)	RCS	96	54	45	NR	NR	NR	NR	NR	NR	NR	NR	UHN	UHN	NR	NR	NR	NR
Sharma *et al.* (8)	P/RCT	43	24	19	42.4 ± 1.8	44.1 ± 2.4	2	4	12|4|8	9|6|4	0	0	NR	NR	100	100	Max 24	Max 24
Verbruggen & Stapert (32)	RCS	21	12	9	75.8 ± 6.2 (64–86)	78.7 ± 6.9 (64–85)	NR	NR	11|1|0	6|2|1	0	1	TLN1	TLN1	92	89	NR	NR

P/NRSIs, prospective/non-randomized studies of interventions; P/RCT, prospective/randomized control trial; RCS, retrospective case series; PTS, patients; aIMN, antegrade intramedullary nailing; rIMN, retrograde intramedullary nailing; NR, not reported; AO, Arbeitsgemeinschaft für Osteosynthesefragen; HLN UOC, humeral locked nail (United Orthopedic Corporation); modGK, modified Grosse Kempf 9 mm tibial nails; EHN, expert humeral nail; MHN, multilock humeral nail; T2, T2 humeral nail (Stryker); TLN1, telescopic locking nail 1 (Stryker); NP, nerve palsy; UHN, unreamed humeral nail (Synthes); FUR, follow-up rate; and MFU, mean follow-up.

* Values are given as mean ± SD (with range in parentheses).

^†^ All radial palsies except brachial plexus injury.

In general, the majority of the included studies made lateral antegrade nail insertion technique (7/9 studies) (5, 6, 8, 22, 25, 32, 33), one study (46) made the entry portal superior for straight nails and lateral for bent nails, and Rommens *et al.* did not report the entry portal for nail insertion they used (34).

All studies assessed functional outcomes, but in such a different way, so we reclassified the information based on PROMs and ROMs to match between studies.

Shoulder functional outcome was based on the CMS system, in which ≤40 points were evaluated as poor or fair functional results, and ROM was graded as poor or fair when there was more than 30 (6) or 50 (5) degree loss of ROM in any direction in comparison with the non-operated side; for the remaining studies, we considered as poor or fair functional results ≤100 degrees of shoulder forward flexion (22, 32). CMS was reported only in three studies (33, 34, 46), but Muckley *et al.* (33) and Metsemakers *et al.* (46) were excluded from analyses because there were insufficient data to separate and extract AG- or RG-specific data for inclusion in our article.

Elbow functional evaluation was performed according to the MEPS system, in which ≤74 points were evaluated as poor or fair functional results, and both Broberg–Morrey score and ROM (graded as poor or fair functional results if there is more than 20 (5, 22, 25, 32) or 30 (6) degree extension loss). MEPS was provided by two studies (5, 34), while Broberg and Morrey score was provided by three studies (32, 33, 46), but Muckley *et al.* (33) and Metsemakers *et al.* (46) did not provide sufficient data to separate and extract AG- or RG-specific data for inclusion in our article.

Sharma *et al.* (8) reported shoulder and elbow stiffness and were considered as poor functional results.

Metsemakers *et al.* (46) measured ROM, but the study was excluded from analyses because we were unable to separate and extract AG- or RG-specific data for inclusion in our article.

The remaining studies (8, 33, 34) did not report ROM results.

None of the studies determined the UCLA shoulder score, ASES score, or DASH score. Neer shoulder score was reported by two studies (5, 32) and was considered an independent outcome.

### Risk of bias assessment

**Figure 2:**
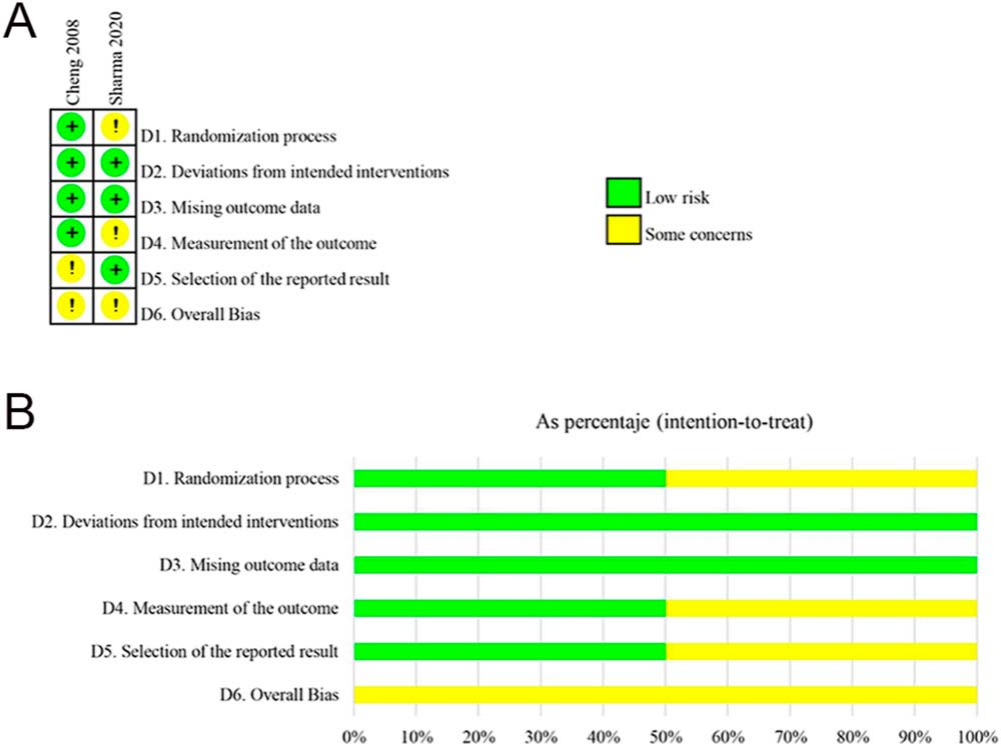
Cochrane risk of bias assessment for RCTs included. (A) Traffic light plot of the risk of bias. D1–D6: bias domain. (B) Weighted bar plot of the distribution of risk of bias judgments within each bias domain.

**Figure 3:**
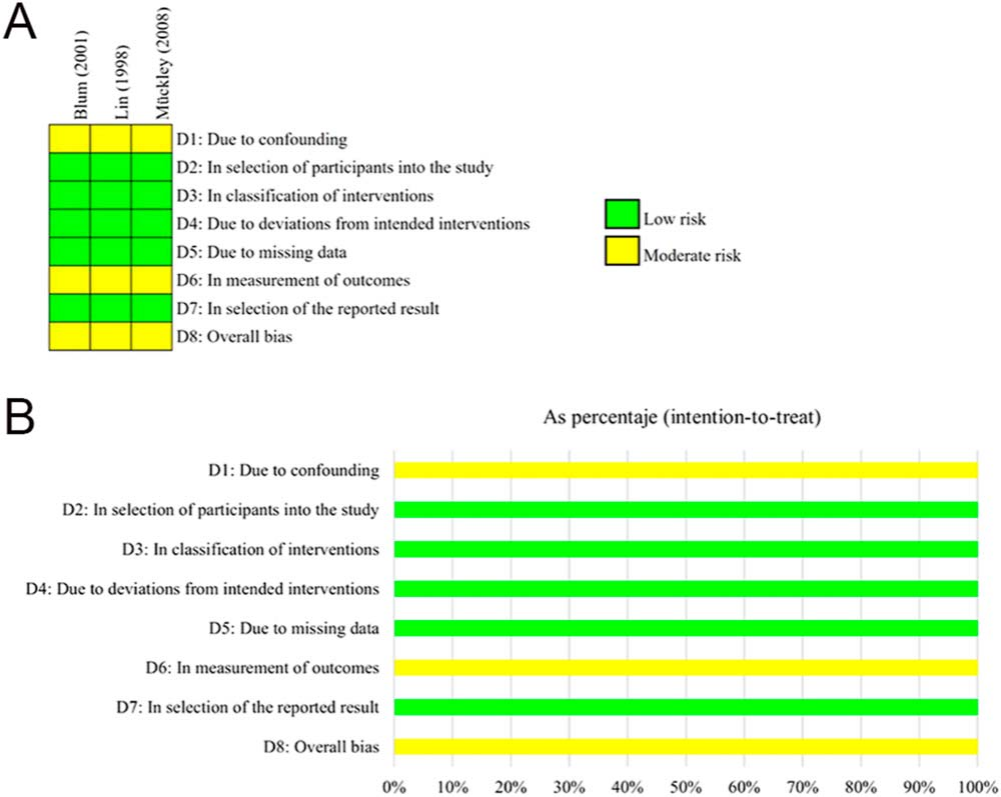
Cochrane risk of bias assessment in non-randomized studies of interventions included. (A) Traffic light plot of the risk of bias. D1–D8: bias domain. (B) Weighted bar plot of the distribution of risk of bias judgments within each bias domain.

The individual risk of bias was assessed according to the study design. The RoB 2 tool was used for the two RCT studies (5, 8) (Fig. 2), and they were deemed to have some concerns regarding risk of bias. According to ROBINS-I (Fig. 3), three prospective NRSIs (6, 25, 33) were evaluated, indicating a moderate risk of bias. Four CS studies (22, 32, 34, 46) were assessed using JBI (Table 2).

**Table 2:** Joanna Briggs Institute (JBI) risk of bias assessment for case series designs included.

Question	Ingman & Waters (22)	Metsemakers *et al.* (46)	Rommens *et al.* (34)	Verbruggen & Stapert (32)
1. Were there clear criteria for inclusion in the case series?	N	Y	N	N
2. Was the condition measured in a standard, reliable way for all participants included in the case series?	Y	Y	Y	Y
3. Were valid methods used for identification of the condition for all participants included in the case series?	Y	Y	Y	Y
4. Did the case series have consecutive inclusion of participants?	Y	Y	Y	U
5. Did the case series have complete inclusion of participants?	Y	N	Y	N
6. Was there clear reporting of the demographics of the participants included in the study?	Y	Y	Y	Y
7. Was there clear reporting of clinical information of the participants?	Y	N	Y	Y
8. Were the outcomes or follow-up results of cases clearly reported?	Y	Y	Y	Y
9. Was there clear reporting of the presenting sites’/clinics’ demographic information?	N	Y	N	N
10. Was statistical analysis appropriate?	N	Y	N	N

Y, yes; N, no; U, unclear.

### Intraoperative outcomes

#### Operative time

**Figure 4:**
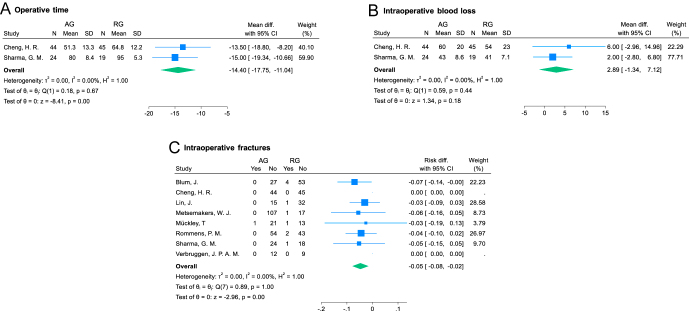
Forest plots for intraoperative outcomes. AG, antegrade group; RG, retrograde group; SD, standard deviation; and CI, confidence interval.

Two studies (5, 8) reporting on 132 procedures (68 in AG and 64 in RG) were included in the analysis of operative time, which was a mean of 66 and 80 min, respectively (significant differences favoring AG: MD = −14.40 (95% CI: −17.75 to −11.04); *I*^2^ = 0%; *P* < 0.001; Fig. 4A).

#### Intraoperative blood loss

Two studies (5, 8) reporting on 132 procedures (68 in AG and 64 in RG) were included in the analysis of intraoperative blood loss, which was a mean of 52 and 48 mm^3^, respectively (differences not significant between groups: MD = 2.89 (95% CI: −1.34 to 7.12); *I*^2^ = 0%; *P* = 0.18; Fig. 4B).

#### Intraoperative fractures

Eight studies (5, 6, 8, 25, 32, 33, 34, 46) reporting on 545 procedures (305 in AG and 240 in RG) were included in the analysis of intraoperative fracture rate. One patient (0.33%) in AG and ten patients (4.16%) in RG developed an intraoperative fracture (significant difference favoring AG: RD = −0.05 (95% CI: −0.08 to −0.02); *I*^2^ = 0%; *P* < 0.005; Fig. 4C). At least four of ten intraoperative fractures developed with retrograde nailing and all that occurred during antegrade nailing were only an observation and were not associated with additional intraoperative fracture fixation or change of fixation strategy.

#### Fluoroscopy time

Fluoroscopy time was not considered in the analysis because it was only provided by one study (5).

### Osseous union outcomes

#### Nonunion

**Figure 5:**
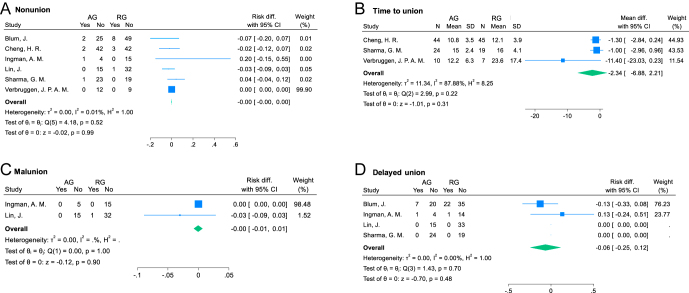
Forest plots for osseous union outcomes. AG, antegrade group; RG, retrograde group; SD, standard deviation; and CI, confidence interval.

Six studies (5, 6, 8, 22, 25, 32) reporting on 305 fractures (127 from AG and 178 from RG) were included in the analysis of nonunion rate. Six patients (4.72%) in AG and 12 patients (6.74%) in RG developed a nonunion (non-significant differences: RD = 0.00 (95% CI: −0.00 to 0.00); *I*^2^ = 0.01%; *P* = 0.99; Fig. 5A). Of these 18 subjects, ten underwent further surgical procedures and seven were not willing to undergo or capable of undergoing a reoperation.

#### Time to union

Three studies (5, 8, 32) reporting on 149 procedures (78 from AG and 71 from RG) were included in the analysis of time to union, which was a mean of 12.7 and 17.2 weeks, respectively (non-significant differences: MD = −2.34 (95% CI: −6.88 to 2.21); *I*^2^ = 87.9%; *P* = 0.31; Fig. 5B).

#### Malunion

Two studies (22, 25) reporting on 68 procedures (20 in AG and 48 in RG) were included in the analysis of malunion rate. No patients in AG (0%) and only one patient (2.08%) in RG developed a malunion (non-significant differences: RD = −0.00 (95% CI: −0.01 to 0.01); *I*^2^ = 0%; *P* = 0.90; Fig. 5C).

#### Delayed union

Four studies (6, 8, 22, 25) reporting on 195 procedures (71 from AG and 124 from RG) were included in the analysis of delayed union rate with eight patients (11.3%) developing a delayed union in AG and 23 patients (18.5%) in RG (non-significant differences: RD = −0.06 (95% CI: −0.25 to 0.12); *I*^2^ = 0%; *P* = 0.48; Fig. 5D).

### Postoperative complications

#### Iatrogenic radial nerve palsy

**Figure 6:**
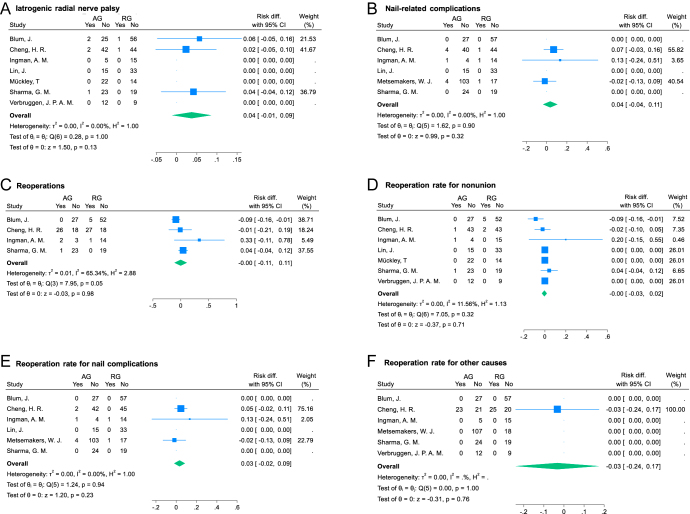
Forest plots for postoperative complications. AG, antegrade group; RG, retrograde group; SD, standard deviation; and CI, confidence interval.

Seven studies (5, 6, 8, 22, 25, 32, 33) reporting on 341 procedures (149 in AG and 192 in RG) were included in the analysis of iatrogenic RNP. Five patients (3.6%) in AG and two patients (1.04%) in RG developed an iatrogenic RNP (non-significant differences: RD = 0.04 (95% CI: −0.01 to 0.09); *I*^2^ = 0%; *P* = 0.13; Fig. 6A). Four iatrogenic RNP recovered completely within two months after surgery, and the evolution of three cases was not reported (6).

#### Superficial infection

Six studies (6, 8, 25, 32, 33, 46) reporting on 357 procedures (207 in AG and 150 in RG) were included in the analysis of superficial infection rate. One patient (0.48%) in AG and two patients (1.33%) in RG developed a superficial infection (non-significant differences: RD = −0.04 (95% CI: −0.15 to 0.07); *I*^2^ = 0%; *P* = 0.48). All three cases responded well to oral antibiotics.

#### Deep infection

Eight studies (5, 6, 8, 22, 25, 32, 33, 34) informed no cases of deep infection. Metsemakers *et al.* (46) reported one case of deep infection, but there were insufficient data to discern group of intervention (AG or RG), so it was excluded from all analyses.

#### Heterotopic ossifications

The presence of heterotopic ossifications was not considered in the analyses because there was only one study (5).

#### Nail-related complications

Six studies (5, 6, 8, 22, 25, 46) reporting on 409 procedures (222 in AG and 187 in RG) were included in the analysis of nail-related complication rate. Nine patients (4.05%) in AG and three patients (1.60%) in RG developed nail-related complications (non-significant differences: RD = 0.04 (95% CI: −0.04 to 0.11); *I*^2^ = 0%; *P* = 0.32; Fig. 6B). See Table 3 for nail-related complication details.

**Table 3:** Nail-related complication details.

Nail-related complication	AG	RG	Total
Screw backout/loosening	4	1	5
Rotatory nail instability (initially locked only at the proximal end)	1	0	1
Fracture displacement for intraoperative fracture or comminution	0	2	2
Fracture distraction due to inadequate compression	1	0	1
Proximal nail protrusion	1	0	1
Fracture displacement due to inadequate reduction	1	0	1
Iatrogenic fracture due to a very short nail segment distal of the fracture (toggling occurred despite the use of locking screws)	1	0	1

AG, antegrade group; RG, retrograde group.

#### Reoperation

Four studies (5, 6, 8, 22) reporting on 236 procedures (100 in AG and 136 in RG) were included in the analysis of reoperation rate. Twenty-nine patients (29%) in AG and 33 patients (24.33%) in RG required a reoperation (non-significant differences: RD = −0.00 (95% CI: −0.11 to 0.11); *I*^2^ = 65.34%; *P* = 0.98; Fig. 6C).

Seven studies (5, 6, 8, 22, 25, 32, 33) reporting on 341 procedures (149 in AG and 192 in RG) were included in the analysis of reoperation rate for nonunion. Three patients (2.01%) in AG and seven patients (3.64%) in RG required reoperation for nonunion (non-significant differences: RD = −0.00 (95% CI: −0.03 to 0.02); *I*^2^ = 11.56%; *P* = 0.71; Fig. 6D).

Six studies (5, 6, 8, 22, 25, 46) reporting on 409 procedures (222 in AG and 187 in RG) were included in the analysis of reoperation rate for nail complications. Seven patients (2.3%) in AG and two patients (0.5%) in RG required reoperation for nail complications (non-significant differences: RD = 0.03 (95% CI: −0.02 to 0.09); *I*^2^ = 0%; *P* = 0.23; Fig. 6E).

Six of nine studies (5, 6, 8, 22, 32, 46) reporting on 382 procedures (219 in AG and 163 in RG) were included in the analysis of reintervention rate for other causes (not due to infection, nonunion or nail complications). Twenty-three patients (10.5%) in AG and 25 patients (15.3%) in RG required reoperation for other causes (non-significant differences: RD = −0.03 (95% CI: −0.24 to 0.17); *I*^2^ = 0%; *P* = 0.76; Fig. 6F). For more details of the type of reintervention carried out by group, see Table 4.

**Table 4:** Type of reinterventions carried out by groups.

Type of reinterventions	AG	RG	Total
Nonunion (*n*)	3	7	10
Bone graft	1	1	2
Bone graft + plate	1	0	1
Plate	0	1	1
Renail + bone graft + wire	1	2	3
Compression with the same nail	0	1	1
Renail + bone graft	0	1	1
Renail	0	1	1
Nail-related complications (*n*)	7	2	9
Screw removal	2	0	2
Insertion of locking distal screw	1	0	1
Cerclage wiring	0	1	1
Plate	2	1	3
Renail	2	0	2
Other causes (*n*)	23	25	48
Tendon transfer for primary radial nerve palsy	2	0	2
Nail removal after consolidation at patient’s request	21	25	46

AG, antegrade group; RG, retrograde group.

Seven studies (5, 6, 8, 22, 25, 32, 33) reported no cases of reintervention for infection, one study (34) reported a wound cleaning for superficial wound infection (34), and another study (46) informed one case of deep infection that required debridement. Both have been excluded from all analyses because we were unable to separate and extract AG- or RG-specific data for inclusion in our article.

### Functional outcomes

#### Neer shoulder score

**Figure 7:**
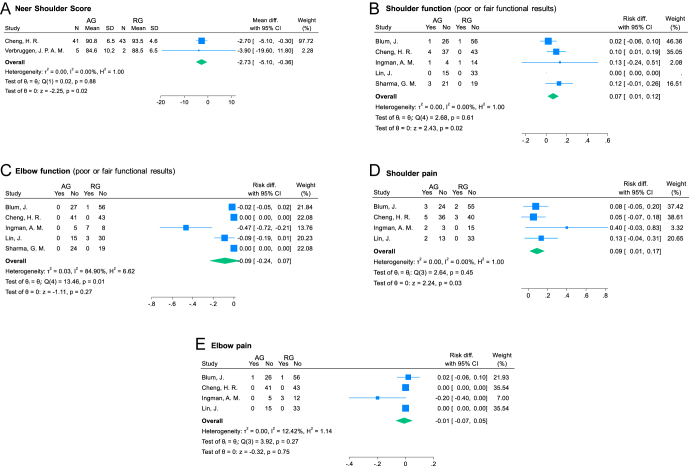
Forest plots for functional outcomes. AG, antegrade group; RG, retrograde group; SD, standard deviation; and CI, confidence interval.

Two studies (5, 32) reporting on 91 procedures (46 in AG and 45 in RG) were included in the analysis of Neer shoulder score. They showed a mean postoperative score of 88 and 91 points, respectively (statistically significant differences favoring RG: MD = −2.73 (95% CI: −5.10 to −0.36); *I*^2^ = 0%; *P* = 0.02; Fig. 7A).

#### Shoulder function

Five studies (5, 6, 8, 22, 25) reporting on 279 procedures (112 in AG and 167 in RG) were included in the analysis of shoulder function. Nine patients (8.04%) in AG and two patients (1.20%) in RG reported poor or fair functional shoulder results (statistically significant differences favoring RG: RD = 0.07 (95% CI: 0.01–0.12); *I*^2^ = 0%; *P* = 0.02; Fig. 7B).

#### Elbow function

Five studies (5, 6, 8, 22, 25) reporting on 279 procedures (112 in AG and 167 in RG) were included in the analysis of elbow function. No patients (0%) in AG and six patients (3.59%) in RG reported poor or fair functional elbow results (non-significant differences: RD = −0.09 (95% CI: −0.24 to 0.07); *I*^2^ = 84.90%; *P* = 0.27; Fig. 7C).

#### Shoulder pain

Four studies (5, 6, 22, 25) reporting on 236 procedures (88 in AG and 148 in RG) were included in the analysis of shoulder pain complaints. Twelve patients (13.6%) in AG and five patients (3.4%) in RG complained of shoulder pain (statistically significant differences favoring RG: RD = 0.09 (95% CI: 0.01–0.17); *I*^2^ = 0%; *P* = 0.03; Fig. 7D).

#### Elbow pain

Four studies (5, 6, 22, 25) reporting on 236 procedures (88 in AG and 148 in RG) were included in the analysis of elbow pain complaints. One patient (1.14%) in AG and four patients (2.70%) in RG complained of elbow pain (non-significant differences: RD = −0.01 (95% CI: −0.07 to 0.05); *I*^2^ = 12.42%; *P* = 0.75; Fig. 7E).

### Evidence quality assessment

The evidence quality obtained by GRADE approach (19, 20, 21) was moderate due to individual study bias for intraoperative fractures, nail-related complications and reinterventions for nail complications and poor for the rest of the outcomes due to individual study bias and imprecision (small sample size), with the exception of the time to union, malunion, rate of reoperations and elbow function, which were extremely low due to the non-applicable heterogeneity in the inconsistency domain (Table 5).

**Table 5:** GRADE working group grades of evidence.

Outcomes	Number of			GRADE assessment						LOE*
	Patients	Studies	aIMN/rIMN	RoB	Inconsistency	Indirectness	Imprecision	OC	Overall COE	
Operative time	132	2	68/64	Serious^†^	Not serious	Not serious	Serious^‡^	None	Θ⨁⨁Θ	Low
Intraoperative blood loss	132	2	68/64	Serious^†^	Not serious	Not serious	Serious^‡^	None	Θ⨁⨁Θ	Low
Intraoperative fractures	542	8	305/240	Serious^†^	Not serious	Not serious	Not serious	None	Θ⨁⨁⨁	Moderate
Nonunion	305	6	127/178	Serious^†^	Not serious	Not serious	Serious^‡^	None	Θ⨁⨁Θ	Low
Time to union	153	3	78/71	Serious^†^	Serious^║^	Not serious	Serious^‡^	None	ΘΘ⨁Θ	Very low
Malunion	68	2	20/48	Serious^†^	Serious^§^	Not serious	Serious^‡^	None	ΘΘ⨁Θ	Very low
Delayed union	195	4	71/124	Serious^†^	Not serious	Not serious	Serious^‡^	None	Θ⨁⨁Θ	Low
Iatrogenic radial nerve palsy	341	7	149/192	Serious^†^	Not serious	Not serious	Serious^‡^	None	Θ⨁⨁Θ	Low
Superficial infection	357	6	207/150	Serious^†^	Not serious	Not serious	Serious^‡^	None	Θ⨁⨁Θ	Low
Nail-related complications	409	6	222/187	Serious^†^	Not serious	Not serious	Not serious	None	Θ⨁⨁⨁	Moderate
Reoperation	236	4	100/136	Serious^†^	Serious^║^	Not serious	Serious^‡^	None	ΘΘ⨁Θ	Very low
For nonunion	341	7	149/192	Serious^†^	Not serious	Not serious	Serious^‡^	None	Θ⨁⨁Θ	Low
For nail complications	409	6	222/187	Serious^†^	Not serious	Not serious	Not serious	None	Θ⨁⨁⨁	Moderate
For other causes	382	6	219/163	Serious^†^	Not serious	Not serious	Serious^‡^	None	Θ⨁⨁Θ	Low
Neer score	110	2	46/45	Serious^†^	Not serious	Not serious	Serious^‡^	None	Θ⨁⨁Θ	Low
Shoulder function	284	5	112/167	Serious^†^	Not serious	Not serious	Serious^‡^	None	Θ⨁⨁Θ	Low
Elbow function	284	5	112/167	Serious^†^	Serious^║^	Not serious	Serious^‡^	None	ΘΘ⨁Θ	Very low
Shoulder pain	241	4	88/148	Serious^†^	Not serious	Not serious	Serious^‡^	None	Θ⨁⨁Θ	Low
Elbow pain	241	4	88/148	Serious^†^	Not serious	Not serious	Serious^‡^	None	Θ⨁⨁Θ	Low

RoB, risk of bias; OC, other consideration; COE, certainty of evidence.

* High certainty: further research is very unlikely to change our confidence in the estimate of effect. Moderate certainty: further research is likely to have an important impact on our confidence in the estimate of effect and may change the estimate. Low certainty: further research is very likely to have an important impact on our confidence in the estimate of effect and is likely to change the estimate. Very low certainty: we are very uncertain about the estimate.

^†^ Moderate risk of bias as assessed with ROBINS-I.

^‡^ Sample size is relatively small (<400).

^§^ Heterogeneity cannot be calculated.

^║^ Heterogeneity >50%.

## Discussion

This study systematically reviewed the efficacy of rigid locked aIMN and rIMN in the management of acute HSFs in non-pediatric patients. The goal was to assess whether the antegrade or the retrograde technique should be used; the hypothesis was that there would be no relevant differences.

It was found that patients treated with aIMN showed advantages over rIMN in surgery time and intraoperative fractures: rIMN took between 11 and 17 min longer than treatment with aIMN, and aIMN demonstrated significantly lower rates (2–8%) of intraoperative fractures than rIMN. However, 40% of intraoperative fractures following rIMN did not require additional surgical procedures. There are no differences in intraoperative bleeding between the use of antegrade and retrograde systems.

There are no differences in bone union outcomes between the use of antegrade and retrograde systems in terms of bone healing rate, delayed healing, nonunion, malunion or time to healing between both techniques.

There are no differences between the use of antegrade and retrograde systems in terms of the presence of iatrogenic nerve paralysis, the rate of superficial infection, complications related to nailing and the rate of reoperation. We have not found sufficient evidence to accept or reject the hypothesis that there are no differences in fluoroscopy time, rate of deep infection or the occurrence of heterotopic ossification between the use of antegrade and retrograde systems. It is important to clarify that this does not necessarily imply that there is no effect, only that data have not provided sufficient evidence to support or reject it.

aIMN had poorer shoulder functional outcomes than rIMN: 8 vs 1% patients reporting poor shoulder function, 14 vs 3% patients reporting residual shoulder pain, and a marginal, non-clinically relevant, 3-point difference in the Neer score. We did not observe significant differences in reported pain and elbow function between the two approaches, although the aIMN approach provides better outcomes for the elbow joint.

As seen in the present study, aIMN seems to be used more frequently than retrograde technique for the treatment of HSFs. One of the reported reasons for hesitance toward using rIMN is caused by fear for supracondylar perioperative fractures during nail insertion and/or reaming (6, 34), and these worries seem to be partially justified. Previous individual studies have reported that if the entry portal is created carefully in rIMN, iatrogenic fractures can be avoided, providing excellent functional results, especially in a young patient population (60). Other studies comparing antegrade with retrograde IMN found that the retrograde approach has a longer learning curve and is technically more demanding due to the nonlinear approach to the medullary canal axis (5), and consideration of the diameter of the humeral shaft is critical to avoid complications. However, avoiding poor shoulder functional outcomes after aIMN requires also an exquisite surgical technique, because of the potential risk of rotator cuff injury and subacromial impingement for the site of entry of aIMN. Preoperative determination of the nail length is a known problem (6). aIMN approach provides better outcomes for the elbow joint, representing a significant advantage, particularly for patients in whom elbow mobility is a priority. These differences should be considered and should also play a role in decision making and informed consent. Complications related to nail removal (22 and 29 patients in AG and RG, respectively) were not analyzed, as no complications occurred in either group.

Although this study found statistically significant differences in some variables between the two techniques, the true clinical relevance of the differences found between both approaches in terms of intraoperative complications requiring additional procedures, union rates, functional results and postoperative complications is probably limited due to the small size of the effect.

### Strength and limitations

As systematic review and MA, findings are dependent on the quality of the studies included (level I, III and IV in this current MA) and the actual number of studies comparing each outcome. Inclusion in the pooled analysis of studies of various levels of evidence and diverse designs (such as RCTs, prospective and retrospective ones) in a systematic review raises serious concerns of potential presence of a high degree of heterogeneity across the recruited material, so the latter had been explored with subgroup analysis. Funnel plots and Egger’s tests were planned to explore publication bias but were not considered as there were fewer than ten studies because they did not have sufficient power to objectively detect bias (17). Forest plots were used instead.

Unfortunately, the literature search found a limited number of studies about rIMN and studies comparing head-to-head aIMN versus rIMN are not recent. Furthermore, not all included studies compared all patient-reported outcomes, which limit the power of the comparisons, and in several studies, some results could not be analyzed because we were unable to separate and extract AG- or RG-specific data for inclusion in our article, data were reported inconsistently, or there were insufficient data for meaningful analysis.

The study by Verbruggen & Stapert (32) reporting on the outcomes of nailing of humerus fractures in the elderly population, although the comparisons between AG and RG were similar, may cause bias of confounding.

In addition, there was variability between studies in terms of the definitions of certain outcomes assessed, such as nonunion, delayed union or malunion, which could have led to over- or underestimation of complication rates; neither there was any standardized point of nail insertion in the antegrade approach, nor postoperative rehabilitation protocol, both of which can affect outcome; and the follow-up duration varied among the studies, so clinical outcomes and the cumulative incidence of complications may differ depending on the follow-up period.

In 2022, Kumar *et al.* (9) published a MA on this topic, but they compared studies with variable implants (both rigid and elastic humeral nails, such as Marchetti–Vicenzi nails (28)) and studies with non-acute HSFs, including delayed healing, nonunion and pathological bone fractures, with time between the injury and surgical management up to 42 weeks (75).

According to our GRADE approach, it is possible that our estimate of effect would be affected by further research because of the low and very low quality of the evidence. However, the best available evidence comparing the outcomes and complications of the management of acute HSFs following rigid locked aIMN and rIMN has been compiled here. High-quality prospective studies performing head-to-head direct comparative studies of aIMN versus rIMN for the management of acute HSFs are required to generate good-quality evidence on the optimal IMN strategy.

## Conclusion

Compared with rIMN, aIMN demonstrated significantly faster operative time and lower rates of intraoperative fractures. Conversely, rIMN demonstrated significantly lower rates of shoulder pain complaints and better shoulder function and Neer score outcomes. The true clinical relevance of the statistical differences found between both approaches is probably limited due to the small size of the effect. No differences in osseous union outcomes and postoperative complications were found. Therefore, based on the evidence available to date, we can conclude that there are no differences between the antegrade and retrograde approaches to diaphyseal humerus fractures in adults that have a real and noticeable impact on medical practice or the patient’s life.

## Supplementary materials



## ICMJE Statement of Interest

The authors declare that there is no conflict of interest that could be perceived as prejudicing the impartiality of the work reported.

## Funding Statement

This work did not receive any specific grant from any funding agency in the public, commercial or not-for-profit sector.
