# Supplementary material for: Rigid locked antegrade versus retrograde intramedullary nailing in treating acute humeral shaft fractures: a systematic review with meta-analysis
Source: EFORT Open Rev. 2026 Feb 4;11(2):131–44. doi: 10.1530/EOR-2024-0136 (PMC12881897; doi:10.1530/EOR-2024-0136)
Supplement: Supplementary file 1 [file supplementary_materials.pdf]

Title: Rigid Locked Antegrade Versus Retrograde Intramedullary Nailing in Treating Acute Humeral Shaft Fractures. A Systematic Review with Meta-Analysis.

Supplementary Materials (SM)

Table 1. PRISMA 2020 Checklist. 2-5
Table 2. PRISMA 2020 for Abstract Checklist. 6
Table 3. Literature search strategy. 7-8

Table 1. PRISMA 2020 Checklist.

Section and Topic	Item #	Checklist item	Location where item is reported
**TITLE**			
Title	1	Identify the report as a systematic review.	1
**ABSTRACT**			
Abstract	2	See the PRISMA 2020 for Abstracts checklist.	SM Table 2
**INTRODUCTION**			
Rationale	3	Describe the rationale for the review in the context of existing knowledge.	3
Objectives	4	Provide an explicit statement of the objective(s) or question(s) the review addresses.	3
**METHODS**			
Eligibility criteria	5	Specify the inclusion and exclusion criteria for the review and how studies were grouped for the syntheses.	4
Information sources	6	Specify all databases, registers, websites, organisations, reference lists and other sources searched or consulted to identify studies. Specify the date when each source was last searched or consulted.	4-5
Search strategy	7	Present the full search strategies for all databases, registers and websites, including any filters and limits used.	SM Table 3
Selection process	8	Specify the methods used to decide whether a study met the inclusion criteria of the review, including how many reviewers screened each record and each report retrieved, whether they worked independently, and if applicable, details of automation tools used in the process.	4-5
Data collection process	9	Specify the methods used to collect data from reports, including how many reviewers collected data from each report, whether they worked independently, any processes for obtaining or confirming data from study investigators, and if applicable, details of automation tools used in the process.	5-6
Data items	10a	List and define all outcomes for which data were sought. Specify whether all results that were compatible with each outcome domain in each study were sought (e.g. for all measures, time points, analyses), and if not, the methods used to decide which results to collect.	5-6
	10b	List and define all other variables for which data were sought (e.g. participant and	5-6

Section and Topic	Item #	Checklist item	Location where item is reported
		intervention characteristics, funding sources). Describe any assumptions made about any missing or unclear information.	
Study risk of bias assessment	11	Specify the methods used to assess risk of bias in the included studies, including details of the tool(s) used, how many reviewers assessed each study and whether they worked independently, and if applicable, details of automation tools used in the process.	5-6
Effect measures	12	Specify for each outcome the effect measure(s) (e.g. risk ratio, mean difference) used in the synthesis or presentation of results.	6
Synthesis methods	13a	Describe the processes used to decide which studies were eligible for each synthesis (e.g. tabulating the study intervention characteristics and comparing against the planned groups for each synthesis (item #5)).	6-7
	13b	Describe any methods required to prepare the data for presentation or synthesis, such as handling of missing summary statistics, or data conversions.	N/A
	13c	Describe any methods used to tabulate or visually display results of individual studies and syntheses.	6-7
	13d	Describe any methods used to synthesize results and provide a rationale for the choice(s). If meta-analysis was performed, describe the model(s), method(s) to identify the presence and extent of statistical heterogeneity, and software package(s) used.	6-7
	13e	Describe any methods used to explore possible causes of heterogeneity among study results (e.g. subgroup analysis, meta-regression).	7
	13f	Describe any sensitivity analyses conducted to assess robustness of the synthesized results.	N/A
Reporting bias assessment	14	Describe any methods used to assess risk of bias due to missing results in a synthesis (arising from reporting biases).	N/A
Certainty assessment	15	Describe any methods used to assess certainty (or confidence) in the body of evidence for an outcome.	6
**RESULTS**			
Study selection	16a	Describe the results of the search and selection process, from the number of records identified in the search to the number of studies included in the review, ideally using a flow diagram.	Figure 1
	16b	Cite studies that might appear to meet the	SM Table 4

Section and Topic	Item #	Checklist item	Location where item is reported
		inclusion criteria, but which were excluded, and explain why they were excluded.	
Study characteristics	17	Cite each included study and present its characteristics.	7-9; Table 1
Risk of bias in studies	18	Present assessments of risk of bias for each included study.	9
Results of individual studies	19	For all outcomes, present, for each study: (a) summary statistics for each group (where appropriate) and (b) an effect estimate and its precision (e.g. confidence/credible interval), ideally using structured tables or plots.	9-16; Table 3-4
Results of syntheses	20a	For each synthesis, briefly summarise the characteristics and risk of bias among contributing studies.	7-9; Figures 2-3; Table 2; SM Figures 1-2 and Table 6
	20b	Present results of all statistical syntheses conducted. If meta-analysis was done, present for each the summary estimate and its precision (e.g. confidence/credible interval) and measures of statistical heterogeneity. If comparing groups, describe the direction of the effect.	9-16
	20c	Present results of all investigations of possible causes of heterogeneity among study results.	N/A
	20d	Present results of all sensitivity analyses conducted to assess the robustness of the synthesized results.	N/A
Reporting biases	21	Present assessments of risk of bias due to missing results (arising from reporting biases) for each synthesis assessed.	N/A
Certainty of evidence	22	Present assessments of certainty (or confidence) in the body of evidence for each outcome assessed.	16; Table 5
**DISCUSSION**			
Discussion	23a	Provide a general interpretation of the results in the context of other evidence.	16-17
	23b	Discuss any limitations of the evidence included in the review.	18-19
	23c	Discuss any limitations of the review processes used.	18-19
	23d	Discuss implications of the results for practice, policy, and future research.	19
**OTHER INFORMATION**			

Section and Topic	Item #	Checklist item	Location where item is reported
Registration and protocol	24a	Provide registration information for the review, including register name and registration number, or state that the review was not registered.	4
	24b	Indicate where the review protocol can be accessed, or state that a protocol was not prepared.	4
	24c	Describe and explain any amendments to information provided at registration or in the protocol.	N/A
Support	25	Describe sources of financial or non-financial support for the review, and the role of the funders or sponsors in the review.	Title page
Competing interests	26	Declare any competing interests of review authors.	Title page
Availability of data, code and other materials	27	Report which of the following are publicly available and where they can be found: template data collection forms; data extracted from included studies; data used for all analyses; analytic code; any other materials used in the review.	Main document

From: Page MJ, McKenzie JE, Bossuyt PM, Boutron I, Hoffmann TC, Mulrow CD, et al. The PRISMA 2020 statement: an updated guideline for reporting systematic reviews. BMJ 2021;372:n71. doi: 10.1136/bmj.n71

For more information, visit: http://www.prisma-statement.org/

Table 2. PRISMA 2020 for Abstract Checklist.

Section and Topic	Item #	Checklist item	Reported (Yes/No)
**TITLE**			
Title	1	Identify the report as a systematic review.	Yes
**BACKGROUND**			
Objectives	2	Provide an explicit statement of the main objective(s) or	Yes

Section and Topic	Item #	Checklist item	Reported (Yes/No)
		question(s) the review addresses.	
**METHODS**			
Eligibility criteria	3	Specify the inclusion and exclusion criteria for the review.	Yes
Information sources	4	Specify the information sources (e.g. databases, registers) used to identify studies and the date when each was last searched.	Yes
Risk of bias	5	Specify the methods used to assess risk of bias in the included studies.	Yes
Synthesis of results	6	Specify the methods used to present and synthesise results.	Yes
**RESULTS**			
Included studies	7	Give the total number of included studies and participants and summarise relevant characteristics of studies.	Yes
Synthesis of results	8	Present results for main outcomes, preferably indicating the number of included studies and participants for each. If meta-analysis was done, report the summary estimate and confidence/credible interval. If comparing groups, indicate the direction of the effect (i.e. which group is favoured).	Yes
**DISCUSSION**			
Limitations of evidence	9	Provide a brief summary of the limitations of the evidence included in the review (e.g. study risk of bias, inconsistency and imprecision).	Yes
Interpretation	10	Provide a general interpretation of the results and important implications.	Yes
**OTHER**			
Funding	11	Specify the primary source of funding for the review.	Yes
Registration	12	Provide the register name and registration number.	Yes

From: Page MJ, McKenzie JE, Bossuyt PM, Boutron I, Hoffmann TC, Mulrow CD, et al. The PRISMA 2020 statement: an updated guideline for reporting systematic reviews. BMJ 2021;372:n71. doi: 10.1136/bmj.n71

For more information, visit: http://www.prisma-statement.org/

Table 3. Literature search strategy.

Database searched	Platform	Query		Records	Records with duplicates
**Medline**	**Ovid**				**1797**
		1	Humeral Fractures/	8014	

		2	humerus/	10763	
		3	fractures bone/	67494	
		4	2 and 3	816	
		5	diaphyseal humeral fracture?.tw.	65	
		6	(middle adj5 humeral adj5 shaft).tw.	30	
		7	humeral shaft fracture?.tw.	850	
		8	1 or 4 or 5 or 6 or 7	8590	
		9	Fracture Fixation Intramedullary/	10835	
		10	Fracture Healing/	14376	
		11	"intramedullary nail*".tw.	7544	
		12	(humer* adj3 nail*).tw.	458	
		13	(bone adj3 (nail* or screw?)).tw.	3519	
		14	((antegrade or retrograde) adj3 (nail* or insertion)).tw.	1474	
		15	((antegrade or retrograde) adj3 intramedullary).tw.	748	
		16	((antegrade or retrograde) adj3 (locked or interlocking)).tw.	167	
		17	9 or 10 or 11 or 12 or 13 or 14 or 15 or 16	29454	
		18	8 and 17	1697	
**Embase**	**Elsevier**				**3495**
		#1	humerus fracture'/exp	15275	
		#2	humerus'/de	13234	
		#3	fracture'/de	104665	
		#4	#2 AND #3	1375	
		#5	diaphyseal humeral fracture$':abti	73	
		#6	middle NEAR/5 humeral NEAR/5 shaft	43	
		#7	humeral shaft fracture$':abti	951	
		#8	#1 OR #2 OR #4 OR #5 OR #6 OR #7	27398	
		#9	intramedullary nailing'/de	16031	
		#10	fracture healing'/de	307	
		#11	intramedullary nail*':abti	8572	
		#12	humer* NEAR/3 nail*	568	
		#13	bone nail*':abti	102	
		#14	bone screw*':abti	1151	
		#15	(antegrade OR retrograde) NEAR/3 (nail* OR insertion)	1943	
		#16	(antegrade OR retrograde) NEAR/3 intramedullary	885	
		#17	(antegrade OR retrograde) NEAR/3 (locked OR interlocking)	195	
		#18	#9 OR #10 OR #11 OR #12 OR #13 OR #14 OR #15 OR #16 OR #17	47805	
		#19	#8 AND #18	3532	
		#20	#8 AND #18 AND [embase]/lim	2915	
**CENTRAL (Cochrane)**	**Wiley**				**6**
		#1	MeSH descriptor: [Humeral Fractures] explode all trees	178	

		#2	MeSH descriptor: [Humerus] explode all trees	97	
		#3	MeSH descriptor: [Fractures Bone] explode all trees	6624	
		#4	#2 and #3	28	
		#5	(diaphyseal humeral fracture?):tiabkw	24	
		#6	(middle NEAR/5 humeral NEAR/5 shaft):tiabkw	3	
		#7	(humeral shaft fracture?):tiabkw	111	
		#8	#1 or #4 or #5 or #6 or #7	267	
		#9	MeSH descriptor: [Fracture Fixation Intramedullary] explode all trees	376	
		#10	MeSH descriptor: [Fracture Healing] explode all trees	552	
		#11	(intramedullary nail*):tiabkw	1031	
		#12	(humer* NEAR/3 nail*):tiabkw	48	
		#13	(bone NEAR/3 (nail* or screw?)):tiabkw	1495	
		#14	((antegrade or retrograde) NEAR/3 (nail* or insertion)):tiabkw	85	
		#15	((antegrade or retrograde) NEAR/3 intramedullary):tiabkw	40	
		#16	((antegrade or retrograde) NEAR/3 (locked or interlocking)):tiabkw	10	
		#17	{OR #9-#16}	2595	
		#18	#8 AND #16	5	
**Total with duplicates**					5298
**Total after duplicates removed**					4601
